# Baicalin regulates stem cells as a creative point in the treatment of climacteric syndrome

**DOI:** 10.3389/fphar.2022.986436

**Published:** 2022-11-02

**Authors:** Qian Wei, Xia Hao, Benson Wui-Man Lau, Shaoxia Wang, Yue Li

**Affiliations:** ^1^ State Key Laboratory of Component-Based Chinese Medicine, Institute of Traditional Chinese Medicine, Tianjin University of Traditional Chinese Medicine, Tianjin, China; ^2^ Department of Rehabilitation Sciences, The Hong Kong Polytechnic University, Hong Kong, Hong Kong SAR, China; ^3^ School of Integrative Medicine, Tianjin University of Traditional Chinese Medicine, Tianjin, China

**Keywords:** climacteric syndrome, baicalin, stem cell, proliferation, differentiation

## Abstract

It is widely acknowledged that the climacteric syndrome negatively affects women’s quality of life and leads to cerebral ischemic injury, osteoporosis and cardiovascular disease. One of the main active ingredients in Radix Scutellariae, Baicalin, has been established to possess a wide range of pharmacological effects and is beneficial in enhancing osteogenic differentiation and cardiovascular disease. Baicalin’s profound metabolic impact on various stem cell populations and their fate specification could improve the efficiency of stem cell therapy for climacteric syndrome. However, Baicalin-mediated processes are complex and many of the underlying mechanisms are not fully fathomed yet. This review aims to shed light on the regulatory role of Baicalin on the diverse behaviors of distinct stem cell populations and provide a good cell source for stem cell therapy to broaden the therapeutic landscape for climacteric syndrome patients.

## Graphical Abstract

**Figure:**
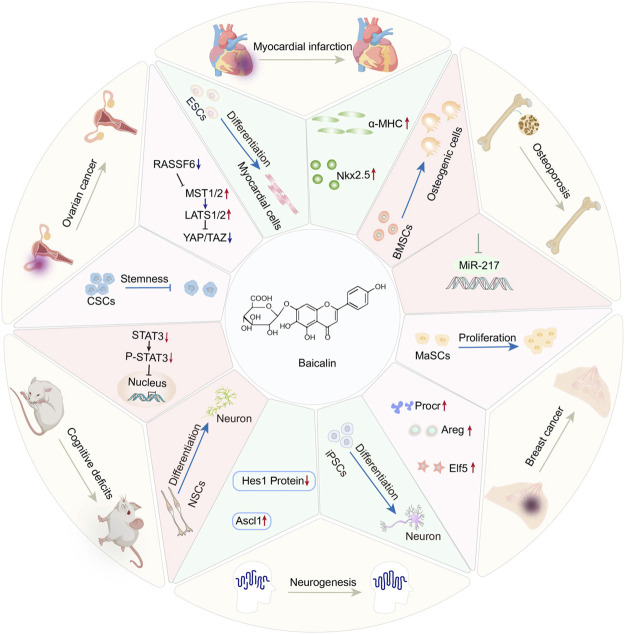
This review summarizes the regulatory role of Baicalin on the diverse behaviors of distinct stem cell populations and emphasizes the potential applications of Baicalin and stem cell therapy in climacteric syndrome.

## Introduction

**FIGURE 1:**
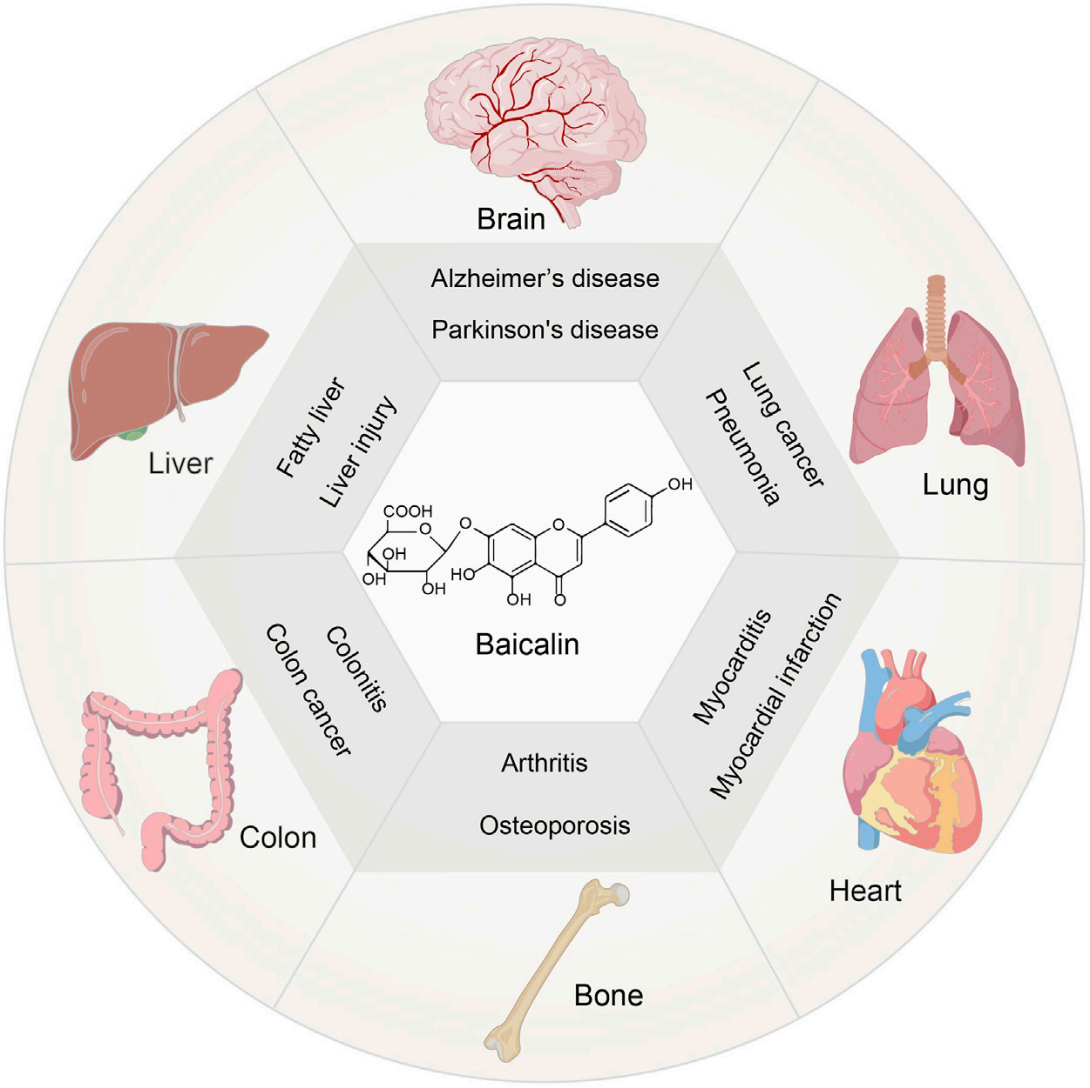
Chemical structure of Baicalin and its extensive pharmacological properties.

Scutellaria baicalensis Georgi has a long history of therapeutic and commercial value in traditional Chinese medicine (Zhao et al., 2019). One of the key constituents is Baicalin, which belongs to the flavonoid family (Zhao Q. et al., 2018). It has been established that Baicalin exerts anti-inflammatory, antioxidant, and anti-apoptotic properties (Guo et al., 2019). Currently, the focus is on studying its pharmacological action before clinical trials for the treatment of various diseases, including liver injury (Shi et al., 2020) and fatty liver(Liu et al., 2020), neurological dysfunction(Jin et al., 2019),lung injury(Zhang H. et al., 2021),osteoporosis(Zhao et al., 2020), inflammation of the colon(Xu et al., 2021) and cardiovascular disease(Xiping et al., 2007) (Figure 1). Moreover, menopause leads to an increase in risk for degenerative diseases and cardiovascular diseases, due to the fluctuation of hormones in women (Brooks et al., 2016), which indicates the possibility of Baicalin in the treatment of climacteric syndrome. Clinical investigations on the treatment of injuries, gum damage, and influenza demonstrate the broad-spectrum pharmacological benefits of baicalin (Table 1). Thus, the therapeutic roles of Baicalin in these severe illnesses emphasizes its potential capacity for management of climacteric syndrome.

**TABLE 1:** Clinical trials of Baicalin in various diseases (www.clinicaltrials.gov).

Drug	Age	Population	Diseases	Formulation type	Status	Clinical trials. Gov identifier	Website link
Baicalin	1–80	30840	Adverse Drug Events	Injection	Completed	NCT01764204	https://www.clinicaltrials.gov/ct2/show/NCT01764204?term=Baicalin&draw=2&rank=1
			Adverse Drug Reactions				
Baicalin	21–62	30	Graft Pain	Ointment	Unknown	NCT03728244	https://www.clinicaltrials.gov/ct2/show/NCT01764204?term=Baicalin&draw=2&rank=2
			Gingival Recession				
Baicalin	2–60	40	Burns	Ointment	Unknown	NCT02737943	https://www.clinicaltrials.gov/ct2/show/NCT01764204?term=Baicalin&draw=2&rank=3

Menopause is a physiological condition that naturally develops in women as they age. Climacteric syndrome is a set of symptoms that often occur throughout the perimenopausal and postmenopausal periods (Wong et al., 2015). Accompanied by mood swings and anxiety and diseases such as osteoporosis(Wang B. et al., 2020), breast cancer (Kabat et al., 2017) and cardiovascular disease (Jaballah et al., 2021) caused by metabolic abnormalities. Cognitive impairment disorders such as Alzheimer’s disease arise because hormone levels have a large impact on the brain (Song et al., 2020). These can negatively burden the quality of life and work efficiency of women. Hormone therapy remains the mainstay of treatment, but it has been associated with risks and side effects (Yang et al., 2022). These findings highlight the need for a new therapeutic approach to the broad spectrum of menopausal symptoms without causing severe side effects. The advent of stem cell technology has offered optimism in treating various diseases in recent decades (Yam et al., 2022). The various mechanisms of action of Baicalin on cells and its ability to regulate various signaling pathways can be used to develop strategies to treat menopause.

Stem cells are self-renewing cells with multi-lineage differentiation potential (McKenzie et al., 2006), which is critical for their involvement in tissue repair and homeostasis (Mi et al., 2022). An increasing body of evidence from recently published studies has demonstrated the efficacy of stem cell therapy in the prevention and treatment of a variety of disorders, including cancer (Steenbruggen et al., 2020), Alzheimer’s disease (Zhu et al., 2020), psoriasis disease (Ali et al., 2020) and dominant optic atrophy (Weiss and Levy, 2019), suggesting that stem cell therapy has great therapeutic potential. Various natural substances are currently available to support stem cell therapy in the treatment of obesity (Hong et al., 2018) and the cure of ischemic cardiomyopathy including myocardial infarction (Han et al., 2019), as well as to increase the treatment effect of osteoporosis and nervous system disorders. As a result, stem cell treatments and natural substances show great promise for tissue regeneration.

Although it is well-established that Baicalin has several positive physiological functions, this paper is focused on how Baicalin regulates physiological mechanisms in embryonic stem cells, neural stem cells and other stem cell populations (Table 2) by demonstrating the physiological mechanisms influencing stem cells for the treatment of different diseases.

**TABLE 2:** The alternations and influences of Baicalin on cellular processes in various stem cells.

Stem cell	Species	Phenotypes	Mechanisms	References
ESC	mouse	Differentiation	NA	Tang et al. (2013)
ESC	mouse	Proliferation	miR-294	Wang, J et al. (2015)
CSC	human	Stemness	YAP	Li et al. (2020)
NSC	mouse	proliferation/differentiation	NA	Zhao, J et al. (2018)
NSC	rat	differentiation	STAT3/bHLH	Li, Y et al. (2012)
C17.2 NSC	mouse	differentiation	Erk1/2	Li et al. (2011)
iPSC	human	differentiation	bHLH	Morita et al. (2015)
MaSC	mouse	Self-renewal	Procr	Chen, M et al. (2021)
BMSC	rat	differentiation	NA	Wang, Q et al. (2020)
HSC	human	differentiation	PPAR	Abbasi et al. (2015)

## Baicalin and embryonic stem cells

Embryonic stem cells (ESCs) have a remarkable capacity for maintaining an undifferentiated condition before differentiating for a long period (Kehat et al., 2001). Since ESCs can proliferate indefinitely and differentiate into any cell type (Xu et al., 2020), they have huge prospects for clinical application (Li et al., 2021). It has been reported that post-menopausal women have a higher risk of cardiovascular disease than when they were younger (Gabriel et al., 2005; Welten et al., 2021). Post-menopausal women also exhibit a greater increase in systolic blood pressure, and total cholesterol and triglyceride levels, as well as low density lipoprotein associated with development of cardiovascular disease, including coronary artery disease. The incidence of myocardial infarction (MI) increases gradually in the post-menopausal women, which is likely to the incidence of men at the age of 80. The result suggest that the onset of myocardial infarction has a sex-specific pathogenesis and seriously affect the quality of life in women (Savonitto et al., 2018). However, the low efficiency of ESCs limits their widespread use. Interestingly, it has been discovered that Baicalin can influence ESC differentiation into cardiomyocytes (Tang et al., 2013) and inhibit cell proliferation (Wang J. et al., 2015). They provide an excellent cell source for myocardial infarction through the differentiation of ESCs, as a novel approach to treating myocardial infarction brought on by female menopause.

**FIGURE 2:**
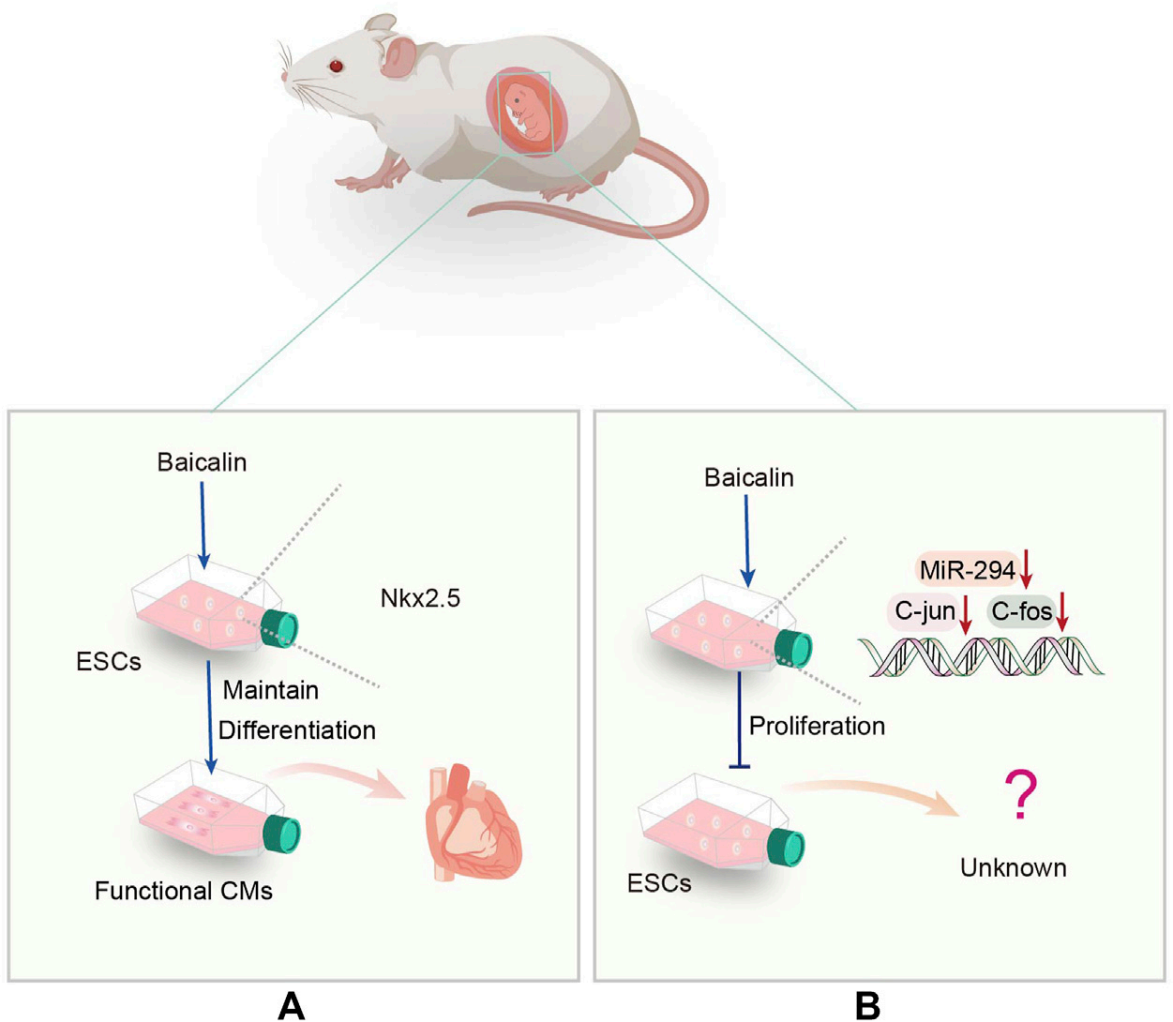
**(A)** Representative scheme illustrating the potential mechanisms underlying the Baicalin regulating the differentiation of ESCs. **(B)** Representative scheme illustrating the potential mechanisms underlying the Baicalin regulation in the proliferation of ESCs.

Myocardial infarction is a common cardiovascular disease of rapid onset (Zhao et al., 2021). Current evidence suggests that although reperfusion can reduce cardiac tissue injury, it increases perfusion injury (Zhang G. et al., 2021). Accordingly, a safer approach is warranted to minimize reperfusion injury in myocardial infarction. Nkx2.5 is an early cardiovascular transcription factor (Thiele et al., 2019), and its specific deletion can lead to cardiac abnormalities(He et al., 2022), suggesting the importance of Nkx2.5 for heart development and growth. Continuous Baicalin treatment can induce functional myocardium formation of embryonic stem cell line D3 by up-regulating the transcription of Nkx2.5 at the intermediate and late stages of differentiation (Tang et al., 2013) (Figure 2A). In addition, the *α* -myosin heavy chain (α-MHC) is a heart-specific gene (Zhu and Lou, 2006) that Baicalin can upregulate. Baicalin stimulates cardiomyocyte differentiation *via* induction of ESCs to restore function after myocardial infarction *via* cell transplantation, providing a new cell source for the treatment of ischemic heart disease, emphasizing the role of Baicalin in the treatment of heart disease. Notably, the Src-Yap1 signaling axis is highly activated in ESCs, and ESC differentiated cells and regulates embryonic stem cell differentiation (Luo et al., 2021). Therefore, it is essential to investigate whether the level of Src-Yap1 fluctuates during treatment with Baicalin on ESC differentiation.

Moreover, it has been demonstrated that the miR-290 family promotes ESC self-renewal by influencing the cell cycle and processes in ESCs (Marson et al., 2008). As a member of this family, miR-294 can promote cell cycle progression and be used in the treatment of myocardial infarction (Borden et al., 2019). Inhibition of miR-294 expression by Baicalin down-regulates the expression of c-Jun and c-Fos genes, leading to an increase in G1 phase, but a decrease in S or G2/M phase of mouse embryonic stem cell line D3 (D3-mESCs), associated with cell proliferation phenotype (Wang J. et al., 2015) (Figure 2B). More importantly, it has been discovered that Baicalin inhibits ESC proliferation. Consequently, more emphasis should be placed on intracellular molecular regulation to improve the current diagnosis and treatment approaches.

In response to hypoxia, Baicalin may activate the HIF1/BNIP3 pathway and produce the upregulated hypoxia-inducible factor 1α (HIF1α), which reduces apoptosis and viability produced by this state, thereby increasing cardiac protection (Yu et al., 2019). Baicalin’s regulatory effect on ESCs highlights that it has huge prospects for myocardial cell differentiation and functional recovery after myocardial infarction. It can inhibit proliferation while promoting differentiation, allowing for better differentiation of ESCs into required cells for clinical application, and offers more therapeutic options for myocardial infarction.

## Baicalin and cancer stem cells

*Cancer* is characterized by aberrant cell development and the potential for metastatic spread and is associated with a high global mortality rate. Reports suggest 19.3 million new cancer diagnoses and 10 million cancer deaths globally in 2020 (Sung et al., 2021). *Cancer* cells are formed partly from the differentiation of cancer stem cells (CSCs), which has become a major research hotspot for treatment in recent decades (Bie et al., 2021). Baicalin has been used to treat a wide variety of cancers since it exhibits anticancer properties in ovarian cancer (Gao et al., 2017). Given the numerous methods of action of Baicalin, the following sections concentrate on the therapeutic benefits of Baicalin on ovarian cancer *via* lowering the stemness of CSCs.

**FIGURE 3:**
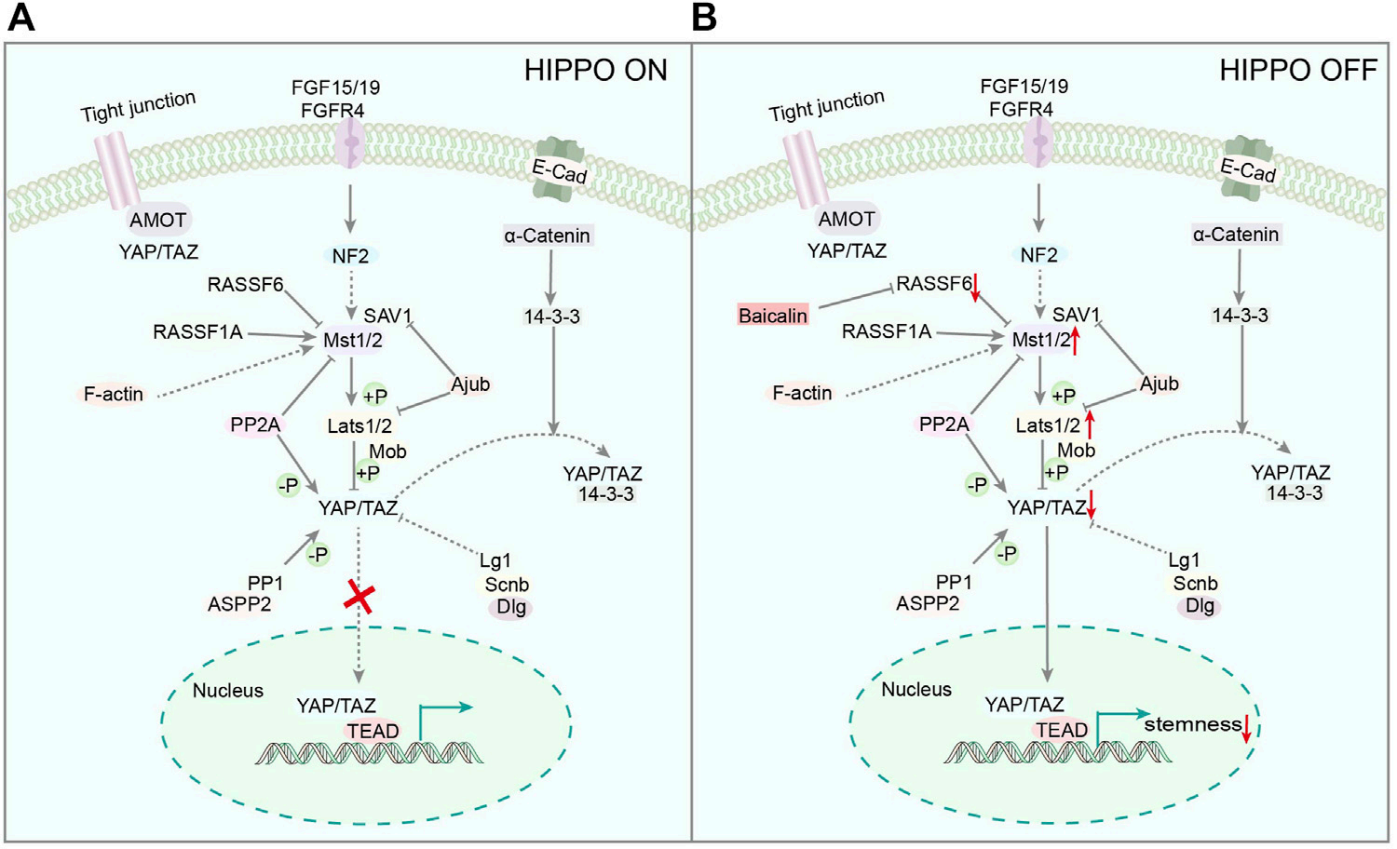
**(A)** Representative scheme illustrating the Hippo Signaling pathway. **(B)** Representative scheme illustrating Baicalin’s role in the proliferation of CSCs by regulating the Hippo Signaling pathway.

It has been established that CSCs is closely related to many signaling pathways, such as YAP (Gao et al., 2020) and Wnt (Tang et al., 2020), which affect the growth and proliferation of cancer cells. The hippocampal/YAP signaling pathway (Hippo/YAP) is a conservative kinase cascade pathway found in *Drosophila melanogaster* (Cordenonsi et al., 2011), containing ste20-like kinase 1/2 (MST1/2) and a large tumor suppressor (Lats1/2), which can be phosphorylated and activated by MST1/2 (Zhou et al., 2020). YAP and transcriptional co-activator (TAZ) are the main downstream effectors of the Hippo pathway, and Lats1/2 can inhibit YAP by direct phosphorylation of S127 (Yu et al., 2012), which play a crucial role in cell fate and maintaining cell stemness (Huang et al., 2020; Quinn et al., 2021). Moreover, Baicalin reduces YAP activity by inhibiting the transcription of RASSF6, a negative regulator of MST1/2 (Li et al., 2020), leading to further inhibition of the stemness of ovarian CSCs (Figure 3), indicating that Baicalin could be utilized to block the YAP signaling pathway *in vitro*. This finding suggests that Baicalin may be utilized to inhibit the YAP signaling pathway. Accordingly, it has huge potential as an anticancer medication to suppress ovarian CSCs.

Furthermore, Baicalin can reportedly increase the chemical sensitivity of cancer cells, reducing drug resistance (Zeng et al., 2020), providing a good auxiliary effect against drug resistance during the cancer treatment process. These discoveries highlight Baicalin’s potential to regulate tumor stem cell growth and how it can benefit humanity by providing the foothold for developing new cancer treatments. As our present understanding of this issue is primarily based on *in vitro* studies, further *in vivo* studies will be required to fully define Baicalin’s ability and promise in the treatment of ovarian cancer.

## Baicalin and neural stem cells

Neural stem cells (NSCs) are undifferentiated cells that can proliferate, self-renew indefinitely, and differentiate into all types of neurons and glial cells (Andreotti et al., 2019). Adult NSCs generate new neurons with active functions throughout their lives, which are integrated into the original neural network to facilitate the development of learning and memory functions (Goncalves et al., 2016). NSCs are crucial in brain development, maturation, and neurogenesis(Di Bernardini et al., 2014). It has been shown that postmenopausal women are more susceptible to neurodegenerative conditions such as ischemic injury and cognitive decline (Ma et al., 2020). Early menopause is associated with an increased risk of stroke (Welten et al., 2021). Moreover, menopause can lead to cognitive issues like Alzheimer’s disease (AD) (Mosconi et al., 2021). These findings highlight the need for new therapeutic approaches to alleviate the symptoms of menopause, thereby reducing cognitive decline and ischemic damage.

Alzheimer’s disease is a degenerative disorder characterized by brain atrophy, loss of neurons, associated with behavioral changes and cognitive decline (Ma et al., 2022). As a common form of dementia, AD affects about 50 million people worldwide and is expected to diagnose a new case every 3 s. However, there is currently no effective treatment available (Liu et al., 2022a). Interestingly, Baicalin has the potential to treat AD through inhibiting Ras-ERK signaling pathway and altering the cell cycle composition ratio, thereby preventing apoptosis caused by Aβ accumulation (Song et al., 2022). In addition, Baicalin can improve synaptic plasticity, mitochondrial fragmentation and dysfunction by inhibiting PDE4 activation in a mouse model of AD (Yu et al., 2022). In addition, Baicalin also affects the differentiation and proliferation of NSCs in AD mouse model (Zhao J. et al., 2018). Accordingly, investigating the effect of Baicalin on NSCs is important to provide novel therapeutic concepts for this patient population. In recent years, research has primarily focused on the role of Baicalin in disease treatment by regulating NSC differentiation. Many signaling pathways, including STAT and ERK, can impact neurodevelopment and NSC fate determination (Boku et al., 2013).

**FIGURE 4:**
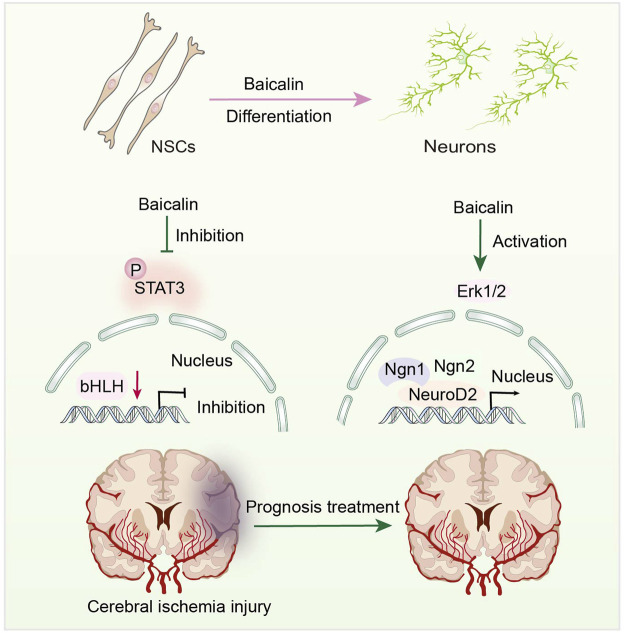
Representative scheme illustrating Baicalin’s role in the proliferation and differentiation of NSCs by regulating the JAK/STAT3 Signaling pathway.

Jak-STAT is an intracellular signal transduction pathway, including Janus Kinases (JAK) and the signal and activator of transcription (STAT). The JAK-STAT pathway is a highly regulated and efficient system that regulates gene expression (Wang T. et al., 2015). STAT3 is involved in NSC differentiation (Boku et al., 2013) and synaptic plasticity (Long et al., 2021). Baicalin can promote neuronal differentiation of embryonic neural stem cells by down-regulating STAT3 phosphorylation (Li Y. et al., 2012) (Figure 4). Baicalin can reportedly ameliorate the usual features of degenerative disorders, such as decreased memory and cognitive function, by promoting differentiation of NSCs. It is widely thought that Baicalin may direct various cells to perform different functions in the central nervous system (CNS). Thus, Baicalin can facilitate the rehabilitation of cognitive impairment caused by aberrant inflammation and age.

Overwhelming evidence substantiates that activating Erk1/2 can increase neurogenesis in C17.2 NSCs (Song et al., 2011; Liu D. et al., 2018), which is employed as a model for assessing the neural differentiation-inducing features of many compounds. Baicalin can stimulate differentiation of C17.2 NSCs by activating Erk1/2 (Li et al., 2011), which controls neural differentiation-related gene expression. Under the effects of Baicalin, the expression levels of NeuroD2, Ngn1, and Ngn2 mRNA are upregulated (Li M. et al., 2012) (Figure 4). Neurogenic proteins (Ngns) and neurogenic differentiation factors (NeuroDs) are pro-transcriptional factors that govern neurogenesis and play a vital role in the development of NSCs into neuronal lineages (Chen W. C. et al., 2019). This finding suggests that Baicalin positively affects NSC differentiation. Moreover, Baicalin has been shown to have no influence on the mRNA expression of split one enhancer (Hes1), Hes5, and DNA binding inhibitor 2 (Id2) (Li M. et al., 2012). These factors inhibit the formation of glial cells while inhibiting neurogenesis (Zhang et al., 2010).

Furthermore, Baicalin has been shown to induce hippocampus regeneration and improve cognitive function following cerebral ischemia injury (Zhuang et al., 2013), providing new insights into the prognosis and treatment of cerebral ischemic injury. These findings account for the ability of Baicalin to increase neurogenesis in clinical trials and provide a novel perspective on NSCs. It is widely thought that Baicalin has the potential to become a small molecule medicine for the regeneration therapy of nervous system illnesses, utilized to alleviate the cognitive impairment and stroke induced by menopause, based on the evaluation of stem cell proliferation and differentiation.

## Baicalin and induced pluripotent stem cells

Induced pluripotent stem cells (iPSCs) are pluripotent, which refer to the cells produced after gene reprogram in somatic cells (Ramotowski et al., 2019). IPSCs have a wide range of sources and are often used to study developmental processes, medical regeneration, and so on (Yamamoto et al., 2021). This phenomenon is notably evident in the nervous system, particularly in light of recent findings on the neuronal differentiation capacity of iPSCs and the benefits of such models for the *in vitro* modeling of AD (Mungenast et al., 2016).

**FIGURE 5:**
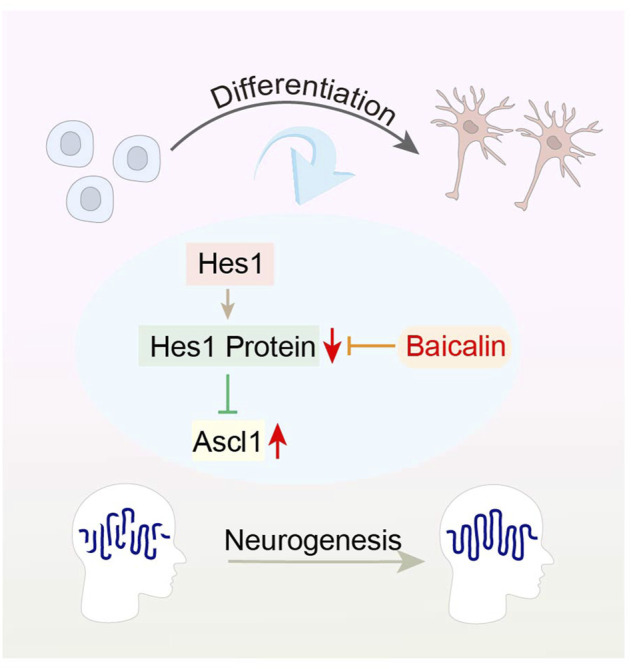
Representative schemes illustrating the potential mechanism of Baicalin regulation in the differentiation of iPSCs.

The basic-helix-loop-helix (bHLH) family of transcription factors, such as Hes1, Ascl1 and Oligo2, play a crucial role in the neural development and fate determination of iPSCs (Wang et al., 2018). Hes1 can induce the expression of glial fibrillary acidic protein (GFAP), which in turn encodes an intermediate filament protein, and inhibit neuronal differentiation by suppressing Ascl1 expression (Ramotowski et al., 2019). Interestingly, Baicalin has been shown to promote neuronal differentiation, but inhibit astroglial differentiation of iPSCs by up-regulating gene expression of Ascl1 and reducing Hes1 protein expression, respectively. The results verify the regulatory effect of Baicalin in bHLH protein family, which is essential for neuronal differentiation of iPSCs (Morita et al., 2015) (Figure 5). Therefore, the ability of iPSCs to differentiate into neurons has made possible therapeutic strategy for neurodegenerative diseases. However, current achievements are only focused on *in vitro* studies, and future research needs on *in vivo* studies in order to obtain more exploratory findings.

## Baicalin and mammary stem cells

Mammary stem cells (MaSCs) have the potential to self-renew and change, developing into luminal progenitors and basal cells, which then differentiate into ducts or alveolar cells (Patwardhan et al., 2014). As a result, MaSCs can sustain mammary gland epithelial and internal environment growth (Jiang et al., 2010) and stimulate mammary gland development and regeneration (Wang et al., 2021). Besides, abnormal regulatory pathways of MaSCs can affect cell activities, leading to breast cancer (Liu R. et al., 2022). Interestingly, it has been reported that hormone levels are associated with the etiology of breast cancer (Ranjan et al., 2021). By 2020, more than 2.3 million women were diagnosed with breast cancer, and 685000 people were killed, exceeding lung cancer-related death for the first time. Although curative rates are dismal, hormone treatment remains the mainstay of therapy (Chen X. et al., 2019). As a result, it is critical to address the process and mechanism of MaSCs in treating breast cancer and mammary gland development, allowing patients to receive better care.

Many natural monomer compounds exhibit hormone-like properties and may be used for medical therapy (Yang et al., 2017). Research has demonstrated that Baicalin can facilitate MaSCs amplification and directly facilitate Protein C receptor (Procr) gene transcription (Chen W. et al., 2021). The Procr gene is essential for the proliferation of MaSCs and the mammary environment (Liu C. et al., 2022). Current evidence suggests that Procr can be used as a surface marker of MaSCs and a therapeutic target for breast cancer (Wang et al., 2019). Several important genes related to basal cell mammary gland development, such as Procr, Areg, Elf5, Socs2, and Bax, are upregulated at the same time, implying that Baicalin promotes mammary gland growth (Chen W. et al., 2021). Baicalin can regulate MaSCs *via* hormone-like activities, providing a good supply of stem cells for stem cell therapy as well as a novel treatment for breast cancer.

## Baicalin and bone marrow mesenchymal stem cells

As pluripotent stem cells, bone marrow mesenchymal stem cells (BMSCs) can differentiate into chondrocytes, osteoblast and fat cells (Khoshsirat et al., 2019). Over the years, BMSCs have been used to treat neurodegenerative diseases (Xue et al., 2019; Liu et al., 2022b) and osteoporosis (Chen M. et al., 2021). As a result, BMSCs represent an important source for the effective treatment of osteoporosis induced by endocrine abnormalities in postmenopausal women. It has been reported that oxidative stress could reduce the osteogenic development of BMSCs during the osteoporosis era (Yang et al., 2021), and even cause BMSCs senescence and apoptosis (Liu Z. et al., 2018). These factors restrict the use of BMSCs in treating neurological illnesses, blood-brain barrier disorders, and bone diseases in the clinic. An efficient antioxidant is accordingly required to minimize oxidative stress and enhance the survival rate of BMSCs.

**FIGURE 6:**
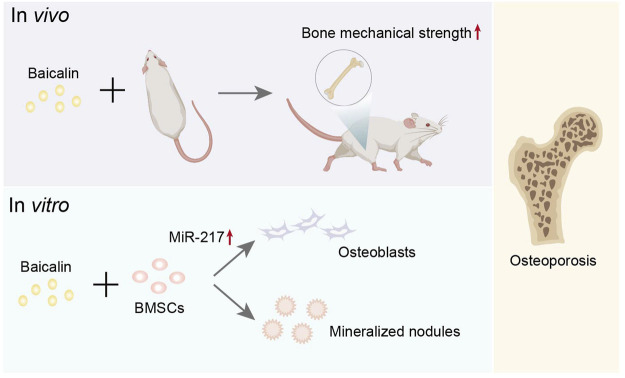
Representative schemes illustrating the potential mechanism of Baicalin regulation in the differentiation of BMSCs.

Interestingly, it is widely thought that Scutellaria baicalensis Georgi has an antioxidant effect (Liau et al., 2019). It has been discovered that BMSCs transplantation might be employed to increase BMSCs transplantation survival rates and bone mechanical strength. Baicalin has been shown to promote BMSC differentiation into osteoblasts and the emergence of mineralized nodules *in vitro* (Zhang et al., 2017), implying that it has an anti-osteoporosis effect (Figure 6). This finding provides compelling evidence for BMSCs transplantation and higher-quality BMSCs for stem cell therapy. Additionally, Baicalin might increase microRNA 217, activate the Wnt/β-catenin and MEK/ERK pathways, and quicken the process of enhancing cell viability and osteogenic differentiation (Wang Q. et al., 2020). Baicalin has huge prospects for application in the treatment of osteoporosis. A study reports that the osteogenic-specific molecules runt-related protein 2 (Runx2) and osteocalcin (Ocn) are expressed more frequently when Baicalin is present (Wang Q. et al., 2020). However, another study demonstrates that miR-217 can bind to Runx2 to prevent rat BMSCs from differentiating into osteoblasts (Zhu et al., 2017). The discrepancy in results may be attributed to different cell lines used in the experiment, and further investigating the effect of miR-217 on osteogenic differentiation is worthwhile. Importantly, Baicalin can increase osteogenic activity and promote bone repair and remodeling, which could have many applications in bone transplantation or osteoporosis.

## Conclusion and future perspectives

This review sought to highlight the effect of Baicalin on stem cells in alleviating several disorders associated with menopause, providing novel insights into the treatment and prevention of climacteric syndrome (Graphical abstract). Current evidence substantiates that Baicalin can be utilized to treat climacteric sickness and control stem cell proliferation, differentiation and self-renewal.

It is widely acknowledged that natural substances like Baicalin have various pharmacological effects, with low toxicity and can cross the blood-brain barrier (Zhao et al., 2022), which offers a basic platform for the treatment of diseases. More study is required to establish the fate of stem cells after treatment with Baicalin, although it has been established that stem cells provide novel insights and therapeutic alternatives for many disorders.

Stem cell therapy has been widely used in recent years and has been clinically used for treating arthritis (Cai et al., 2023), brain injury (Jiao et al., 2022) and other diseases. However, its disadvantages cannot be ignored, such as immunologic rejection and poor cell viability (Chen et al., 2022). As it turns out, Baicalin can enhance stem cell efficacy, serve as a good source of stem cells for stem cell therapy, and augment the regulatory function of stem cells. Baicalin and stem cell therapy can work synergistically to extend the drug’s scope of use and therapeutic potential. In order to unlock the therapeutic potential of Baicalin at a deeper level, an interdisciplinary approach is required to uncover the cellular and molecular mechanisms of Baicalin in these processes and the associated pathological pathways.

Indeed, various shortcomings of Baicalin should be addressed before its implementation for the treatment of climacteric illness. Baicalin, for example, has been demonstrated *in vivo* to limit the proliferation of hematopoietic stem cells (HSCs) in a concentration-dependent way (Abbasi et al., 2015) After hydrolysis into baicalein *in vivo* (Wang et al., 2018), baicalein activates ERK by reducing MKP3 aquaporin and triggering the Nrf-2 pathway, causing upregulation of cytokines (Patwardhan et al., 2014) and boosting the number of HSCs even further. Further research is warranted on the *in vivo* transformation of the two and the combined regulation of stem cells. Furthermore, Baicalin has poor water solubility and a limited bioavailability (Mi et al., 2021), limiting its future clinical applicability. To increase medication usage, dosage formulations such as cyclodextrin inclusion and hydrogel are currently being explored (Li et al., 2018; Wang et al., 2022). Therefore, further dosage optimization represents a major future challenge, and the therapeutic effects of different doses still need to be explored. Prior studies on the molecular pharmacology of Baicalin on stem cells showed that both could be utilized to treat diseases that are stem cell-related. Treatment for illnesses connected to climacteric syndrome has benefited from all these investigations. We anticipate our assessment will generate fresh perspectives for further debate on Baicalin and stem cell research.

## Abbreviations

AD, Alzheimer’s disease; BMSCs, bone marrow mesenchymal stem cells; CSC, cancer stem cells; CNS, central nervous system; Id2, DNA binding inhibitor two; ESCs, embryonic stem cells; HSC, hematopoietic stem cell; Hippo/YAP, hippocampal/YAP signaling pathway; iPSCs, human-induced pluripotent stem cells; HIF1α, hypoxia-inducible factor 1α; JAK, Janus Kinases; Lats1/2, large tumor suppressor; MaSCs, Mammary stem cells; NSCs, Neural stem cells; NeuroDs, neurogenic differentiation factors; Ngns, Neurogenic proteins; Ocn, osteocalcin; Procr, Protein C receptor; Runx2, runt-related protein two; STAT, signal and activator of transcription; Hes1, split one enhancer; MST1/2, ste20-like kinase 1/2; TAZ, transcriptional co-activator; α-MHC, α-myosin heavy chain.
