# Supplementary figures and images for: Baicalin regulates stem cells as a creative point in the treatment of climacteric syndrome
Source: Front Pharmacol. 2022 Nov 2;13:986436. doi: 10.3389/fphar.2022.986436 (PMC9666758; doi:10.3389/fphar.2022.986436)

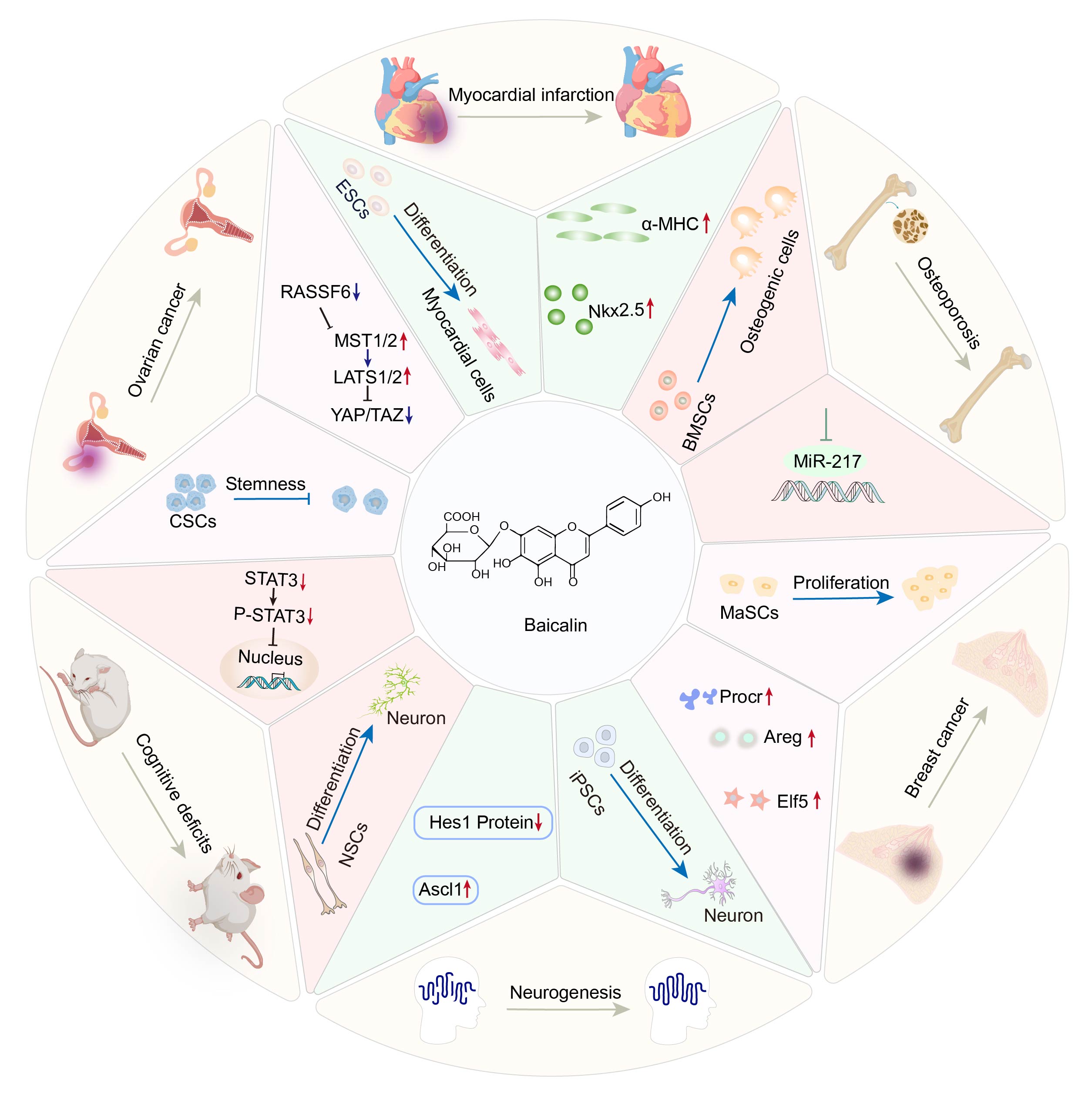

Supplement: Supplementary file 1 [file Image1.JPEG]
